# Photoinduced Dynamics of [N(C_3_H_7_)_4_]_2_[Cu_4_Br_6_] Thin Films with Dual Self‐Trapped Exciton Emission and Negative Thermal Quenching

**DOI:** 10.1002/cphc.202401143

**Published:** 2025-03-25

**Authors:** Domenic Gust, Alexander Merker, Konstantin Moritz Knötig, Kawon Oum, Thomas Lenzer

**Affiliations:** ^1^ Department Chemistry and Biology Physical Chemistry 2 Faculty IV: School of Science and Technology University of Siegen Adolf-Reichwein-Str. 2 57068 Siegen Germany

**Keywords:** Molecular salts, Halocuprates, Ultrafast laser spectroscopy, Self-trapped excitons, Triplet state

## Abstract

Organic‐inorganic halocuprates(I) form a promising class of light‐emitting materials with high photoluminescence (PL) quantum yield. However, the understanding of their emission properties and the PL mechanism is still limited. Here, we investigate thin films of bis(tetrapropylammonium) hexa‐μ‐bromo‐*tetrahedro*‐tetracuprate(I), [N(C_3_H_7_)_4_]_2_[Cu_4_Br_6_], which has a zero‐dimensional (0D) molecular salt structure containing [Cu_4_Br_6_]^2−^ ions. The compound shows a bright orange PL, consisting of two bands, with a quantum yield of about 95 % at room temperature. An analysis of the temperature‐dependent width of the two emission bands provides large Huang‐Rhys factors of 81 and 33, which are assigned to two self‐trapped exciton states (denoted as STE1 and STE2) with different excited‐state structures of the anion. For both STE bands, a decrease of the lifetime from 82 to 32 μs over the temperature range 80–323 K is accompanied by an increase of the PL band integral, indicating an unusual negative thermal quenching process. The microsecond lifetimes are consistent with a phosphorescence process. Broadband transient absorption experiments from the femto‐ to the microsecond regime provide a time constant for S_1_→T_1_ intersystem crossing (ISC) step of 490 ps and time scales for the cooling processes in S_1_ and T_1_.

## Graphical abstract

Temperature‐dependent time‐resolved photoluminescence (PL) and transient absorption spectroscopy track the generation and decay of two self‐trapped exciton states (STE1 and STE2) in an [N(C_3_H_7_)_4_]_2_[Cu_4_Br_6_] thin film after photoexcitation. This efficient zero‐dimensional halocuprate(I) emitter shows a quite rarely observed increase of the PL intensity with temperature in the range 80–323 K, also known as negative thermal quenching.

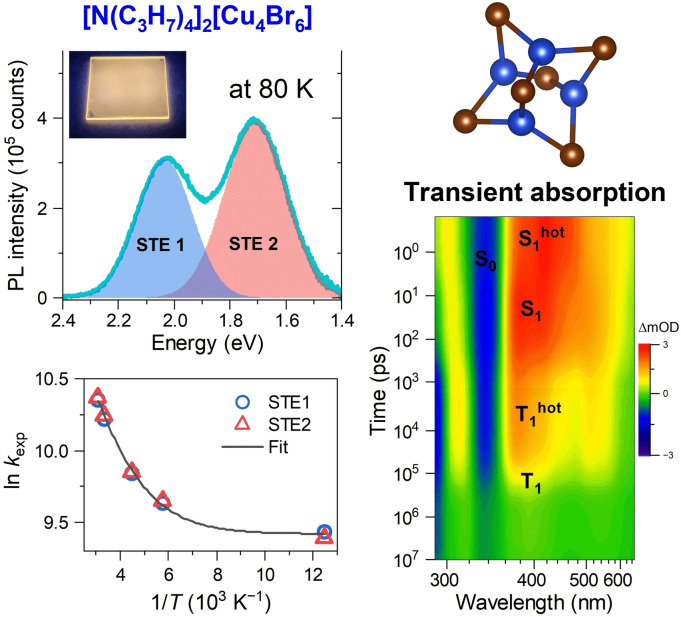

## Introduction

Halocuprates(I) with organic countercations of the general formula A_(*n*–*m*)/*c*_Cu_*m*_X_*n*_ (A=organic cation, X=halide, *m*<*n*, *c*=charge of the organic cation) have recently come under intense scrutiny because many members of this class have a high photoluminescence quantum yield exceeding 90 %.[^1^, ^2^] This property renders them attractive materials for the application in light‐emitting diodes (LEDs), with the additional benefit of the wide abundance of copper and the typically low toxicity of these compounds, in particular when compared with potential competitors, such as lead‐based perovskite‐type materials. Through variation of the organic countercation, e. g. by using different types of alkylammonium and alkylphosphonium derivatives, the structure of the halocuprate(I) anion chromophore can be widely adjusted, encompassing zero‐dimensional (0D), one‐dimensional (1D) and two‐dimensional (2D) structures.^[3]^ This structural variation has a distinct impact on the photophysical properties. For instance, a wide tunability of the emission wavelength is possible,[^4^, ^5^] leading to the formation of blue,[^6^, ^7^] green[^8^, ^9^, ^10^, ^11^] and orange‐red emitters.[^4^, ^10^, ^11^, ^12^, ^13^] Tunability of the emission spectrum can be even achieved for the same anion chromophore. For instance, looking at different halocuprates(I) incorporating the [Cu_4_Br_6_]^2−^ anion, the compounds [P(C_2_H_5_)_4_]_2_[Cu_4_Br_6_], [N(C_4_H_9_)_4_]_2_[Cu_4_Br_6_], [N(C_4_H_9_)_3_CH_3_]_2_[Cu_4_Br_6_] and [N(C_3_H_7_)_4_]_2_[Cu_4_Br_6_] show yellow‐orange to orange‐red emission, with peak wavelengths of 601, 605, 623 and 664 nm.[^4^, ^11^, ^13^] Even double‐peak emission was observed for [P(C_2_H_5_)_4_]_2_[Cu_4_Br_6_].^[11]^ What can be already concluded from the previous measurements for these four [Cu_4_Br_6_]^2−^‐based compounds is that there is no simple correlation of the position of their peak emission with the average Cu−Cu distance in the ground state structure of the anion. As an example, in the aforementioned series of compounds the average Cu−Cu distances in the distorted Cu_4_ tetrahedra of [Cu_4_Br_6_]^2−^ are 2.713, 2.744, 2.743 and 2.726 Å, showing no systematic trend. This finding suggests that instead the excited‐state relaxation of the anion after photoexcitation in the crystal environment is much more important for the emission properties of halocuprates(I). In fact, drastic structural changes of the S_1_ and T_1_ structures have been reported previously, for example in the study of [CH_3_NH_3_]_4_[Cu_2_Br_6_].^[9]^

In addition to the high PL quantum yield and the wide tunability of the photoluminescence, other photophysical parameters of these emitters need to be considered, which are crucial for their performance. One important point is the color purity of the emission, which is connected to the width of the emission band. For display applications, LEDs with a narrow emission of the primary colors blue (ca. 460 nm), green (ca. 525 nm) and red (ca. 675 nm) would be ideal.^[14]^ In addition, the excited‐state lifetime of the emitter must be compatible with the refresh rate of a display and should also not compromise the LED saturation threshold by slow repopulation of the excited states. Therefore, values in the range 5–50 μs are preferred for phosphorescent compounds.^[14]^

The [N(C_3_H_7_)_4_]_2_[Cu_4_Br_6_] system, first synthesized by Asplund and Jagner,^[15]^ is an efficient halocuprate(I) triplet emitter and exhibits a PL quantum yield of 95–97 % in the single crystal.[^4^, ^12^] For the related tetrabutylammonium‐based system, [N(C_4_H_9_)_4_]_2_[Cu_4_Br_6_], Feng, Wang, Sun and co‐workers reported a quantum yield of 84 %.^[16]^ Here, we will focus on thin films of [N(C_3_H_7_)_4_]_2_[Cu_4_Br_6_], because these have the highest relevance when it comes to applications in LEDs, requiring film deposition either by wet‐chemical methods, such as spin‐coating, or evaporation. This compound displays unusual dual emission consisting of two broad PL bands. We provide a detailed characterization of the photophysical properties of [N(C_3_H_7_)_4_]_2_[Cu_4_Br_6_] using steady‐state and time‐resolved emission spectroscopy over a wide temperature range (80–323 K). These experiments are complemented by an extended UV–Vis broadband transient absorption study on the femto‐ to microsecond time scale to identify the intermediate states of [N(C_3_H_7_)_4_]_2_[Cu_4_Br_6_] populated en route to the emissive triplet species, as well as their lifetimes.

## Results and Discussion

### X‐Ray Diffraction of Thin Films

**Figure 1:**
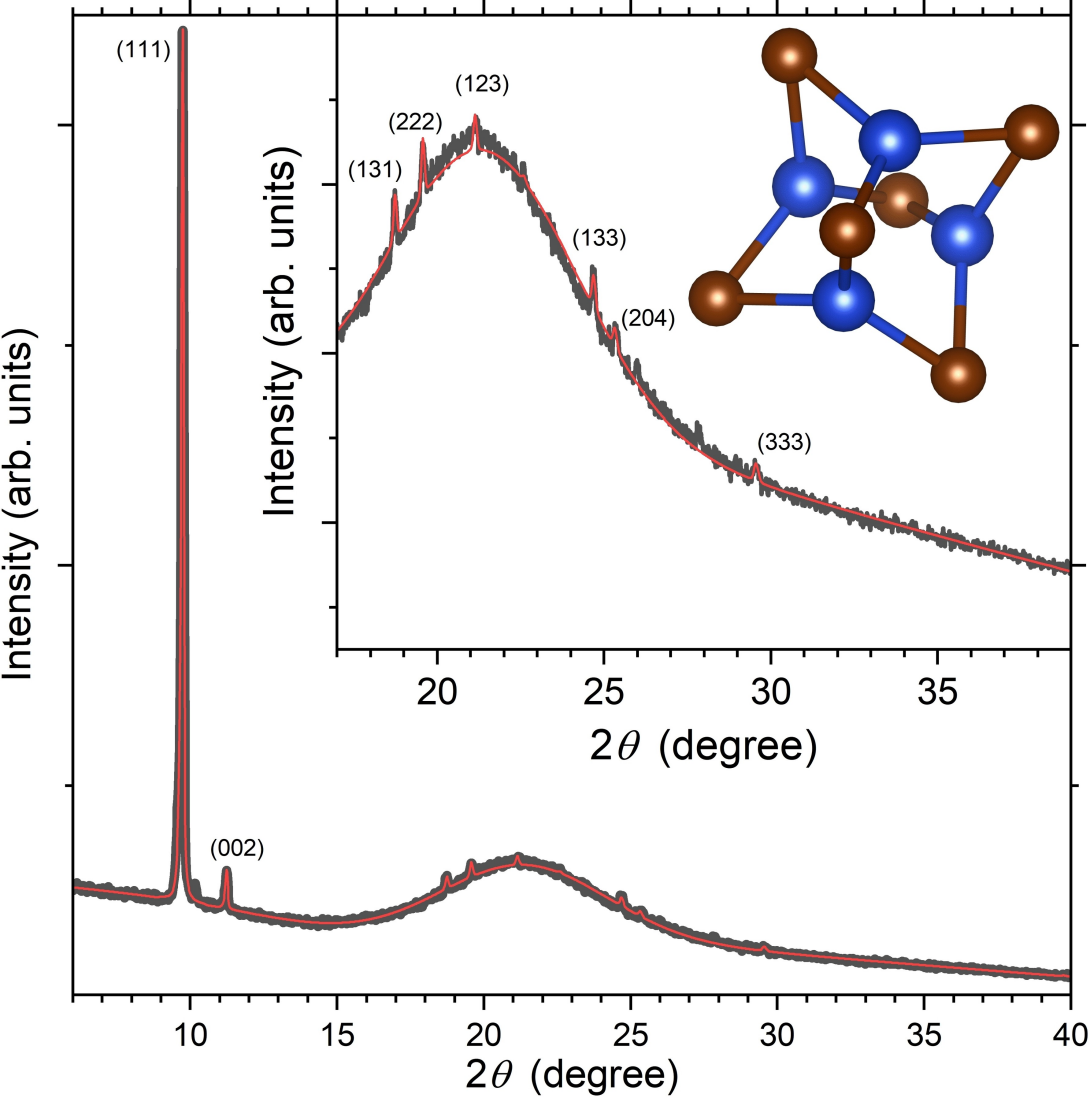
X‐ray diffractogram of an [N(C_3_H_7_)_4_]_2_[Cu_4_Br_6_] thin film (black line) compared with the simulation result based on a Rietveld refinement approach (red line). Inset: Enlargement of the data in the 2*θ* range 17–39° with a ball‐and‐stick representation of a single [Cu_4_Br_6_]^2−^ anion (copper(I): blue, bromide: bronze, view along the *c* axis of the crystal). Note that the diffraction peaks in the inset at 26.0° and 27.8° match well with several known reflexes of this compound, but we could not reproduce their intensity in the texture simulation. The peak at 10.1° cannot be assigned to [N(C_3_H_7_)_4_]_2_[Cu_4_Br_6_] and also not to any of the precursors or [N(C_3_H_7_)_4_][CuBr_2_]. It is possibly an instrumental artifact.

Figure 1 shows the X‐ray diffractogram of an [N(C_3_H_7_)_4_]_2_[Cu_4_Br_6_] thin film (black line) together with a fit obtained from a Rietveld refinement (red line).^[17]^ [N(C_3_H_7_)_4_]_2_[Cu_4_Br_6_] crystallizes in the tetragonal space group *P*4_2_/*n* (no. 86) with the unit cell parameters *a*=*b*=15.687 Å and *c*=15.724 Å. The structural parameters are in good agreement with those previously determined by Asplund and Jagner (*a*=*b*=15.708 Å and *c*=15.709 Å)^[15]^ and by Chen et al. (*a*=*b*=15.5454 Å and *c*=15.5737 Å).^[4]^

Substantial texture effects of the film were observed, as for instance indicated by the pronounced (111) reflex in the diffractogram, consistent with a preferential growth direction of the film. The compound has a zero‐dimensional (0D) molecular salt structure containing [Cu_4_Br_6_]^2−^ anions embedded in a framework of tetrapropylammonium cations. Each anion consists of an octahedron of bromide ligands which contains a tetrahedron of trigonal coordinated copper(I),^[15]^ as shown in the inset of Figure 1.^[18]^

### Optical and Scanning Electron Microscopy (SEM)

**Figure 2:**
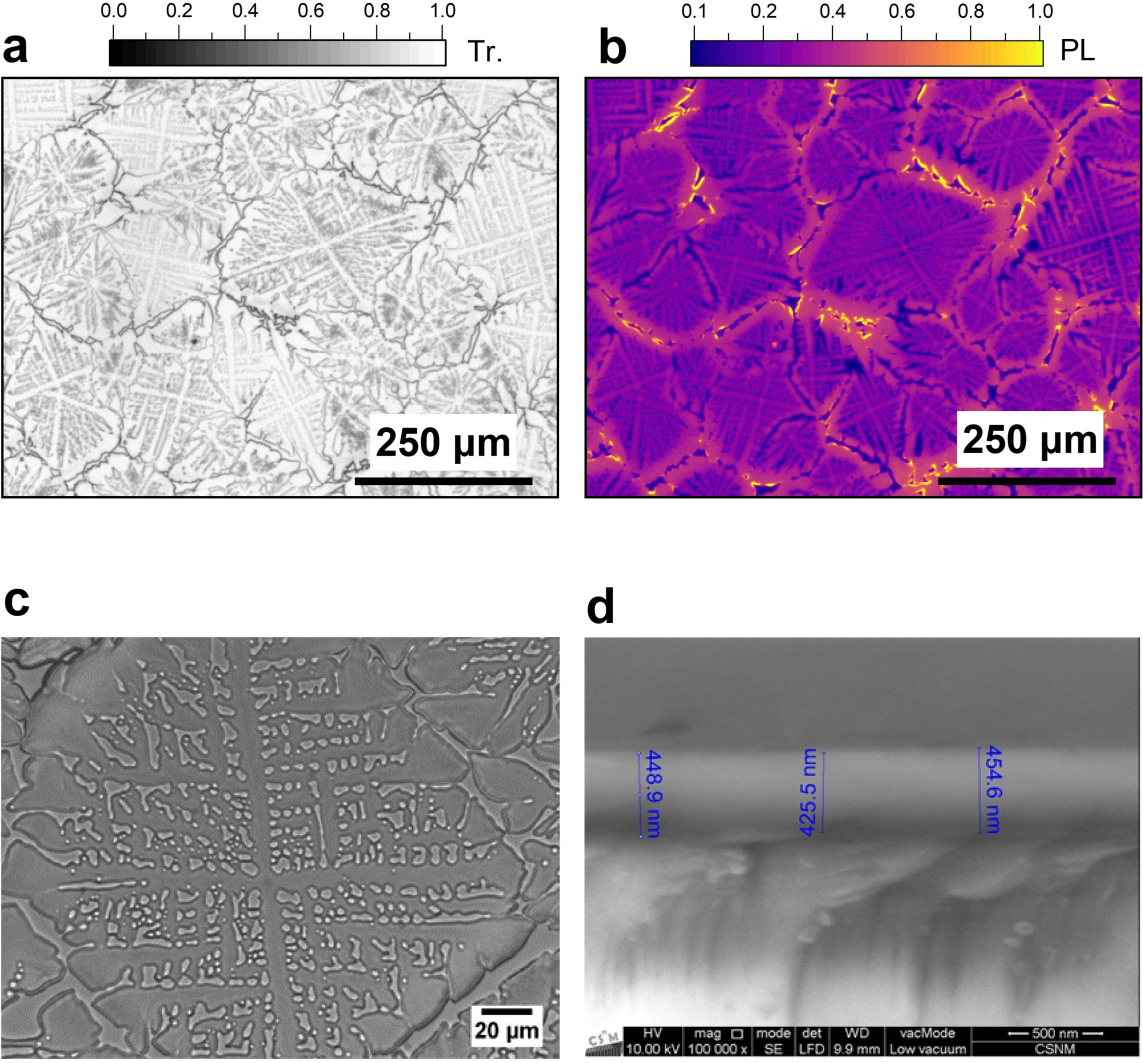
Images from optical and SEM microscopy. (a) Image from optical microscopy in transmission for an [N(C_3_H_7_)_4_]_2_[Cu_4_Br_6_] thin film (scale bar: 250 μm). (b) Photoluminescence microscope image at the same magnification and position as in panel a (excitation wavelength: 330 nm, detection using a 500 nm longpass filter). (c) Image from optical microscopy in transmission (scale bar: 20 μm). (d) Cross‐sectional view from SEM at a magnification of 10^5^ with the film thickness indicated in blue at three different positions (scale bar: 500 nm).

Figure 2 contains optical transmission and PL microscope images as well as a scanning electron microscope image of an [N(C_3_H_7_)_4_]_2_[Cu_4_Br_6_] thin film on glass. The optical measurements in transmission at different magnifications depicted in Figure 2a,c show a dense microcrystalline film with a crystallite size in the range 50–200 μm. The PL microscope image in Figure 2b, recorded for the same region as in panel a, shows the emission from these microcrystalline grains. Increased PL can be seen at the grain boundaries. This is likely due to waveguiding effects inside of the microcrystallites. The PL emitted sideways from different positions propagates by total internal reflection and emerges at the grain boundaries leading to pronounced edge emission. Based on the cross‐sectional SEM picture of the film in Figure 2d, we estimate a film thickness of about 440 nm.

### Steady‐State Absorption and Photoluminescence

**Figure 3:**
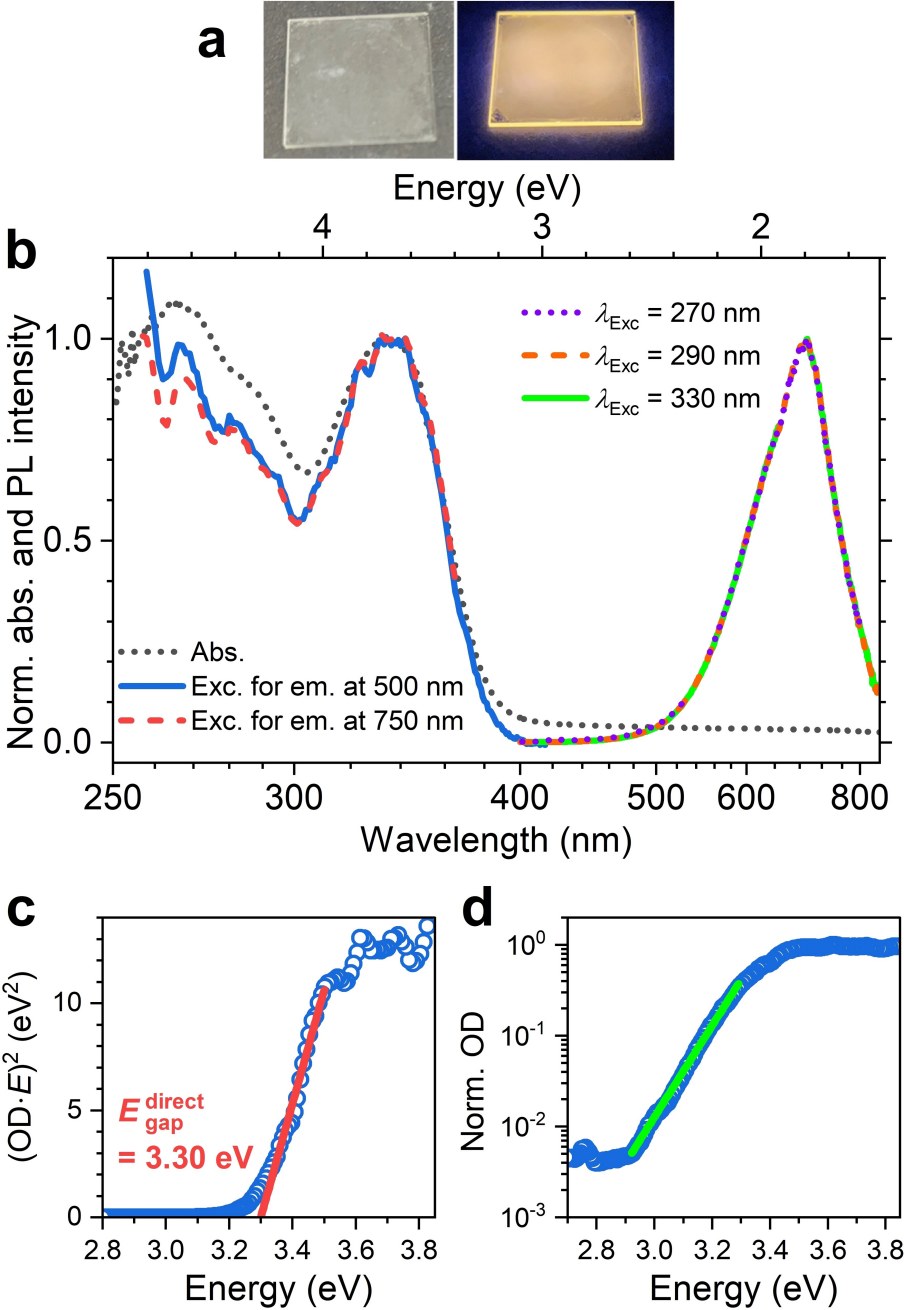
Photographs and steady‐state optical spectra at 298 K. (a) Two photographs of an [N(C_3_H_7_)_4_]_2_[Cu_4_Br_6_] thin film under daylight (left) and the orange emission when irradiated by LED light at 365 nm (right). (b) Normalized steady‐state absorption (black dotted line) and two excitation spectra (blue solid line: emission at 500 nm, dashed red line: emission at 750 nm). Normalized PL spectra recorded at 270 nm (violet dotted line), 290 nm (orange dash‐dotted line) and 330 nm (green solid line). (c) Tauc fit (red solid line) of the excitation spectrum recorded at 500 nm assuming a direct band gap. (d) Excitation spectrum recorded at 500 nm shown on a semilogarithmic scale including a fit of the Urbach tail (green line) based on eq. (1).

The two photographs in Figure 3a show the thin film of [N(C_3_H_7_)_4_]_2_[Cu_4_Br_6_] on quartz in daylight (left) and under irradiation by an LED with a center wavelength of 365 nm (right) demonstrating the orange emission originating from the colorless, slightly opaque film. Figure 3b contains the normalized steady‐state absorption, PL and excitation spectra at 298 K. The steady‐state absorption spectrum (black dotted line) shows a maximum at about 335 nm (*E*_abs_^max^=3.70 eV), corresponding to the transition from the valence band (VB) to the conduction band (CB) using a band‐model terminology. The position of the absorption band is in reasonable agreement with the DFT/TDDFT calculations of Latouche, Gautier and co‐workers, which find the first absorption peak of the isolated [Cu_4_Br_6_]^2−^ anion at ca. 370 nm.^[19]^ Considering the 0D molecular salt structure and using the terminology employed for molecules, this band can be also assigned to the S_0_→S_1_ single–singlet transition of the [Cu_4_Br_6_]^2−^ anion. Another absorption band rises below 300 nm suggesting absorption into higher excited singlet states. These spectral features are well reproduced by the excitation spectra (blue solid line and red dashed line, respectively), which were recorded at an emission wavelength of 500 and 750 nm, respectively. Because the excitation spectra detected on both wings of the emission band are identical, one can conclude that the emission band originates from the same excited singlet state.

Relevant parameters of the band structure were extracted from a Tauc plot for a direct band gap, i. e. a plot of (OD⋅*E*)^2^ vs. *E*, with OD being the optical density (=absorbance), as shown in Figure 3c. Because the absorption spectrum of the film shows a long tail above 400 nm, which arises from light scattering of the opaque film (Figure 3a), we employed the essentially background‐free excitation spectrum for this plot instead (blue circles). Using the straight part of the band edge region we extrapolate a direct band gap energy *E*_gap_^direct^ of 3.30 eV (376 nm). Closer inspection of the Tauc plot reveals a weak absorption tail below the band gap energy. This behavior was analyzed further using an Urbach plot which is shown in Figure 3d. The Urbach energy tail in the absorption spectrum can be described by the following exponential function:[^20^, ^21^]

(1) $$\mathrm{OD}={\mathrm{OD}}_{0}\exp\left(-\frac{E-E_{t}}{E_{U}}\right)$$

Here, OD_0_ is a scaling factor, *E*_t_ describes the energetic position of the Urbach tail, and *E*_U_ is the Urbach energy.^[22]^ *E*_t_ was estimated as the value of the direct band gap (*E*_t_≈*E*_gap_^direct^=3.30 eV), yet the exact value has practically no impact on the fitted Urbach energy.

The Urbach tail describes the exponential decay of the spectrum very well (green line in Figure 3d). We obtain a value of 86 meV for *E*_U_, which is in the same range as the values previously obtained for the silver iodobismuthate AgBi_2_I_7_ (70 meV)^[22]^ or amorphous silicon (67 meV).^[23]^ The quite large value of *E*_U_ suggests some crystalline disorder. In contrast, typical Urbach energies for more ordered semiconductors, such as lead halide perovskites, monocrystalline silicon, CdTe, GaAs, GaN or InP are in the range 11–25 meV.[^24^, ^25^, ^26^, ^27^]

The normalized PL spectrum at 298 K in Figure 3b (violet dotted, orange dash‐dotted and green solid lines for excitation at 270, 290 and 330 nm, respectively) has a peak maximum at 1.80 eV (675 nm). The shape of the PL spectrum does not change by varying the excitation wavelength. Its full width at half maximum (FWHM) is about 0.45 eV, and a large experimental Stokes shift Δ*E*_Stokes,exp_ of *E*_abs_^max^−*E*_PL_^max^=1.90 eV (peak‐to‐peak) is obtained. Using the direct band gap value from the Tauc plot, one obtains *E*_gap_^direct^−*E*_PL_^max^=1.50 eV. Such a large energy difference between the emission and absorption bands is not compatible with emission from a direct band gap which should appear in the wavelength range 380–420 nm (see also Figure 3b).

### Temperature‐Dependent Steady‐State Photoluminescence Spectra

The broad PL band with a large Stokes shift likely originates from self‐trapped excitons in the triplet state, as will be discussed in detail below. Such self‐trapping is induced by the structural relaxation of the crystal lattice in the vicinity of the photoexcited halocuprate(I) [Cu_4_Br_6_]^2−^ ions. The anions themselves also experience a structural relaxation in the excited state. Both effects lead to the substantial red‐shift of the photoluminescence.

**Figure 4:**
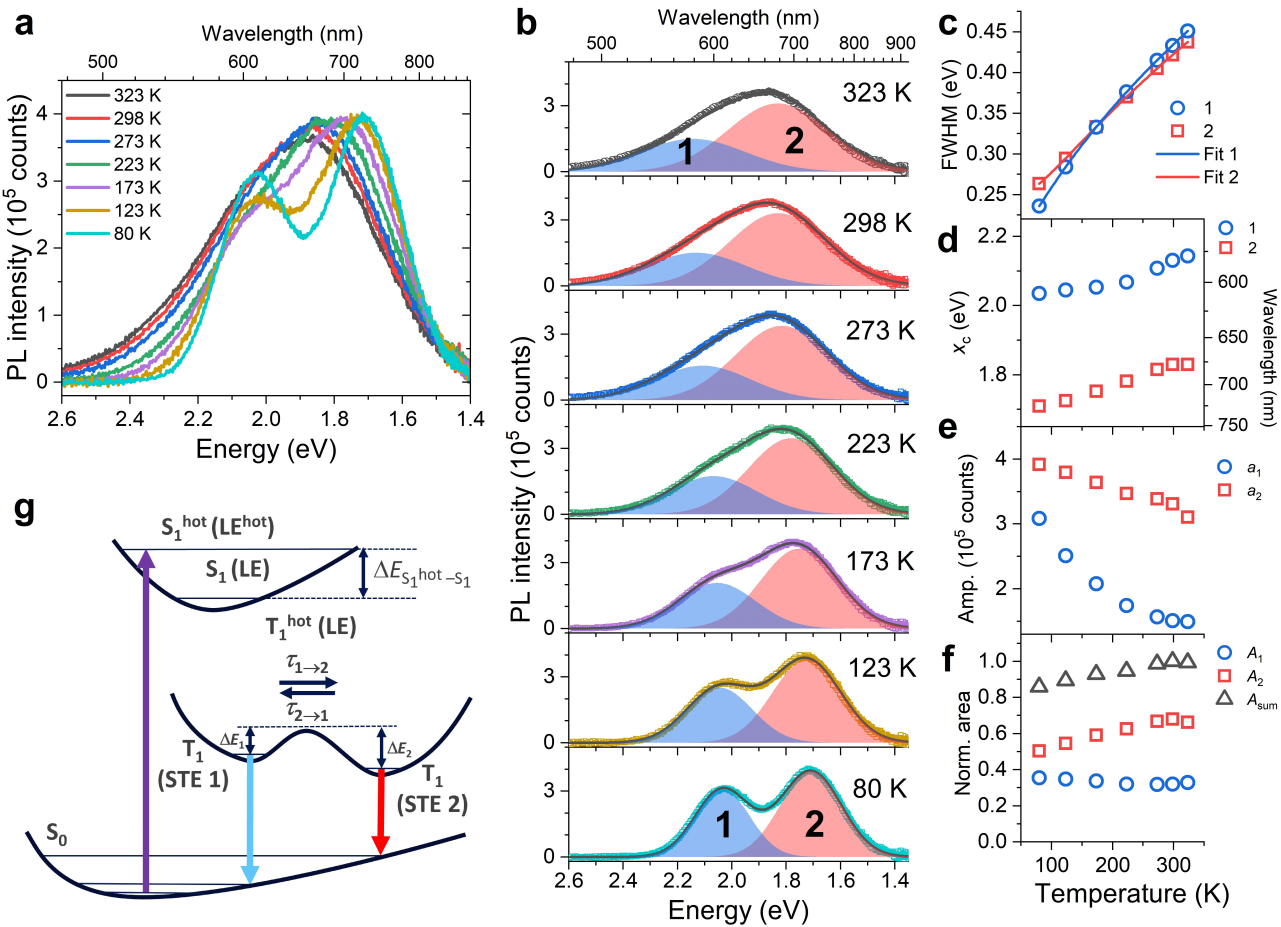
Analysis of the temperature‐dependent photoluminescence spectra of an [N(C_3_H_7_)_4_]_2_[Cu_4_Br_6_] thin film. (a) Spectra recorded over the temperature range 80–323 K. (b) Results of the peak analysis. Each spectrum was fitted by a sum (solid black line) of two Gaussian functions, marked as “1” (blue‐filled areas) and “2” (red‐filled areas), respectively. The fit parameters are summarized in panels (c)–(f): full‐width at half maximum (FWHM), peak center (*x*_c_), peak amplitude (*a*) and peak areas (*A*_1_, *A*_2_, *A*_sum_=*A*_1_+*A*_2_), respectively, as a function of temperature. STE1 band (blue circles), STE2 band (red squares). In panel f, the total peak area (*A*_sum_=*A*_1_+*A*_2_) is indicated by black triangles (areas scaled, so that *A*_sum_ at 298 K is normalized). (g) Scheme of the electronic states involved and the proposed emission mechanism. Abbreviations of states: LE (localized exciton) and STE (self‐trapped exciton).

Temperature‐dependent PL experiments were carried out to characterize the STE emission mechanism of the [N(C_3_H_7_)_4_]_2_[Cu_4_Br_6_] thin film in more detail. Figure 4a,b shows the PL spectra obtained at different temperatures in the range 80–323 K excited by an LED with a center wavelength of 365 nm. The data clearly show that the emission spectrum consists of two distinct bands, in the following labeled as STE1 (high‐energy band, blue) and STE2 (low‐energy band, red). The contributions of the two bands to the whole spectrum can be most clearly distinguished at low temperatures. As shown in Figure 4b, the spectral shape is well reproduced by the sum of two Gaussian functions. The individual emission bands are labeled as “1” (blue‐filled area) and “2” (red‐filled area) for the higher energy and lower energy parts, respectively. A peak analysis provided parameters for the FWHM, peak center (*x*_c_), peak amplitude (*a*), and band area (*A*). These values are summarized for the Gaussian bands in Figures 4c–f as a function of temperature. Figure 4g shows our proposed emission mechanism for this thin‐film system.

At 80 K, the two PL bands can be clearly discerned, with band maxima at 2.03 eV (609 nm, STE1) and 1.71 eV (725 nm, STE2). When the temperature is increased to 323 K, both PL bands become broader. As shown in Figure 4c, the value for the full width of half maximum Δ*E*_FWHM_ increases from 236 meV to 450 meV for the STE1 band and from 263 meV to 437 meV for the STE2 band, with a slightly smaller slope for the STE2 band. In the following, we apply a one‐dimensional configuration coordinate model for the singlet ground state S_0_ and the respective T_1_ triplet STE states involved. A detailed description of the model and relevant parameters are provided in the Supporting Information (Supporting Note 1). The change in width for each of the two STE bands can be quantified in terms of the total Huang‐Rhys factor *S* and the phonon energy *E*_ph_, representing an average over the spectral density of electron–phonon coupling, by using the equation[^28^, ^29^, ^30^]

(2) $$\Delta E_{\mathrm{FWHM}}=\;\sqrt{8\ln\left(2\right)}\;\sqrt{S}\;E_{\mathrm{ph}}\;\sqrt{\coth\left(\frac{E_{\mathrm{ph}}}{2k_{B}T}\right)}\;$$

where *k*_B_ is the Boltzmann constant. The resulting fits are shown in Figure 4c. We obtained Huang‐Rhys factors *S* of 81.2 and 33.2 and phonon energies *E*_ph_ of 8.1 meV and 18.1 meV for the STE1 and STE2 bands, respectively. The large Huang‐Rhys factors suggest that this system is in the regime of strong electron–phonon coupling.^[28]^ At such large *S* values, the line shape of an STE band, which essentially represents the phonon wing of the emission spectrum with practically no intensity in the zero‐phonon line, is expected to have a structureless and only slightly distorted Gaussian shape.[^28^, ^31^] In the present case, it was indeed sufficient to fit each STE band by a single Gaussian function to arrive at a very good fit. The Stokes shift between the peak of the S_0_→T_1_ absorption band and the peak of the T_1_→S_0_ emission band can be calculated as:^[28]^

(3) $$\Delta E_{\mathrm{Stokes},\mathrm{Huang}-\mathrm{Rhys}}=\;2\;E_{\mathrm{ph}}\;S$$

with resulting values Δ*E*_Stokes,Huang‐Rhys_ of 1.32 eV and 1.20 eV for STE1 and STE2, respectively. Next, we compare these model values with the experimentally determined peak‐to‐peak Stokes shift Δ*E*_Stokes,exp_ for STE1 and STE2 at 298 K of 1.57 eV and 1.87 eV, respectively (extracted from Figure 4b). The differences between these experimental values and the values obtained from the Huang‐Rhys fits are 0.25 eV (triplet STE1) and 0.67 eV (triplet STE2), resulting in an average difference of 0.46 eV.

It is important to realize at this point that the absorption band observed in the experimental spectrum is the optically allowed S_0_→S_1_ transition, whereas the forbidden S_0_→T_1_ transition is assumed in the configuration coordinate model for the triplet STEs. The experimentally measured Stokes shift is considerably larger than Δ*E*_Stokes,Huang‐Rhys_ of eq. (3), because Δ*E*_Stokes,exp_ contains the following two additional energy contributions: (a) the energy difference $\Delta E_{S_{1}^{\mathrm{hot}}-S_{1}}$ between the initially photoexcited “hot” Franck‐Condon state S_1_^hot^ and the relaxed localized‐exciton (LE) state S_1_ (both of singlet character) which is released to the lattice, and (b) additional thermal energy released to the lattice after the S_1_→T_1_ ISC process has finished. The term “localized exciton” is justified for such a molecular salt system, because the excitation is primarily located on a single molecular anion chromophore.

As described in more detail in the Supporting Information (Supporting Note 1), one can also determine the S_1_−T_1_ energy splitting $\Delta E_{S_{1}-T_{1}}$ (i. e. the energy difference of the zero phonon lines of S_1_ and T_1_) for both states. They are 0.51 eV for STE1 and 0.87 eV for STE2. Previous TDDFT calculations for another zero‐dimensional halocuprate(I) system, [CH_3_NH_3_]_4_[Cu_2_Br_6_], found that $\Delta E_{S_{1}-T_{1}}$ for the isolated [Cu_2_Br_6_]^4−^ anion is 0.34 eV.^[9]^ Assuming a similar singlet–triplet splitting $\Delta E_{S_{1}-T_{1}}$ for the isolated [Cu_4_Br_6_]^2−^ anion, this value suggests that the energy difference of 0.17 eV (STE1) and 0.53 eV (STE2) is due to self‐trapping, i. e. stabilization of the anion due to relaxation of the [N(C_3_H_7_)_4_]^+^ cations surrounding the anion.

When the temperature increases from 80 K to 323 K, the STE bands not only become broader, but also shift to higher energy (shorter wavelength), as shown by the change in peak position (*x*_c_) in Figure 4d. Over this temperature range, we observed a blue shift from 2.04 eV (609 nm) to 2.15 eV (578 nm) of about 0.11 eV (31 nm) for STE1, and from 725 nm to 678 nm of about 0.12 eV (47 nm) for STE2, respectively. The effect most likely arises from a thermal expansion of the lattice accompanied by a change in electron–phonon coupling.[^32^, ^33^]

Furthermore, over the range 80–323 K, the increase in the width of the STE bands is accompanied by a decrease of their peak amplitude (Figure 4e). As shown in Figure 4f, the total peak area (*A*_sum_=*A*_1_+*A*_2_, with the value of *A*_sum_ at 298 K normalized to 1) increases from 86 % to 99 % in the range 80–273 K, suggesting an increase of the overall PL quantum yield over this temperature interval. Looking at the individual contributions of STE1 and STE2, the band integral for the lower‐energy STE2 band increases in the range 80–273 K, thereby overcompensating the slight decrease of the higher‐energy STE1 band area. Over the range 273–323 K, the total band integrals and also the individual band integrals of STE1 and STE2 remain approximately constant. Assuming that there is a temperature‐dependent equilibrium between STE1 and STE2 (with the time constants *τ*_1→2_ and *τ*_2→1_, compare Figure 4g), this could indicate that in the temperature range 80–223 K the population distribution between STE1 and STE2 shifts more towards STE2 with increasing temperature, leading to the increase in the band integral for STE2. Alternatively, assuming that STE1 and STE2 decay independently, the temperature‐dependent changes in the band integrals of STE1 and STE2 could indicate different changes in the temperature dependence of the nonradiative rate constants of STE1 and STE2. We would like to point out that one should be cautious in interpreting the exact values of the band integrals in too much detail, as certain deviations may occur because the absorption spectrum changes its shape slightly with temperature and therefore the photoexcitation by the UV‐LED light source (center wavelength 365 nm) in our experiment might lead to a slightly different initial number density of the excited‐state chromophores.

At the end of this section, we would like to discuss possible origins of the two STE emission bands of [N(C_3_H_7_)_4_]_2_[Cu_4_Br_6_]. TDDFT calculations of gas‐phase [Cu_4_Br_6_]^2−^ by Latouche, Gautier and co‐workers found two stable structures in the triplet state which differ substantially from the ground‐state structure shown in the inset of Figure 1. One of these structures preserves the Cu_4_ tetrahedron, but has a strongly distorted Br_6_ octahedron, whereas the other structure features a substantially distorted, “opened up” Cu_4_ tetrahedron, with one strongly elongated Cu−Cu bond. The emission transitions of these two triplet state minima were calculated as 488 nm and 850 nm, respectively.[^19^, ^34^] Although there is a significant difference between these calculated triplet emission energies for the isolated anion and the values found here in a crystalline environment (Figure 4b), it would indeed be reasonable to assume that the emission bands of STE1 and STE2 are associated with two structural minima in the T_1_ state. Changes in the excited‐state Cu−Cu bond lengths in the anion therefore have a profound impact on the emission wavelength of the halocuprates(I). The degree of stabilization of the different triplet‐state structures will be also influenced by the type of countercation. It is already known that changes in the organic countercation induce changes in the Cu−Cu distances in the electronic ground state of [Cu_4_Br_6_]^2−^.[^4^, ^11^, ^13^] Therefore, similar effects are expected in the excited state.

Our results for [N(C_3_H_7_)_4_]_2_[Cu_4_Br_6_] can be also compared with those for other halocuprate(I) systems that exhibit multiple STE emission bands. For example, in the case of the bromocuprate(I) [NH_3_−(CH_2_)_4_−NH_3_][Cu_2_Br_4_], Wang et al. observed two STE emission bands with peaks at 460 nm (STE1) and 605 nm (STE2), with a change from dual emission (STE1 and STE2) to exclusively blue emission (STE1 only) upon increasing the temperature from 80 to 340 K.^[35]^ In this case, significantly different excitation spectra were obtained for the two STE bands, in contrast to our results for [N(C_3_H_7_)_4_]_2_[Cu_4_Br_6_] (Figure 3b). In another example of an iodocuprate(I) system featuring 4‐dimethylamino‐1‐ethylpyridinium as counterion, Song et al. observed a thermal transformation between two different types of 1D anion chains, from [Cu_2_I_3_]^−^ (blue STE emission) to [Cu_4_I_6_]^2−^ (yellow STE emission) via a concerted copper atom migration process.^[36]^

### Time‐Resolved Broadband Photoluminescence at 298 K

**Figure 5:**
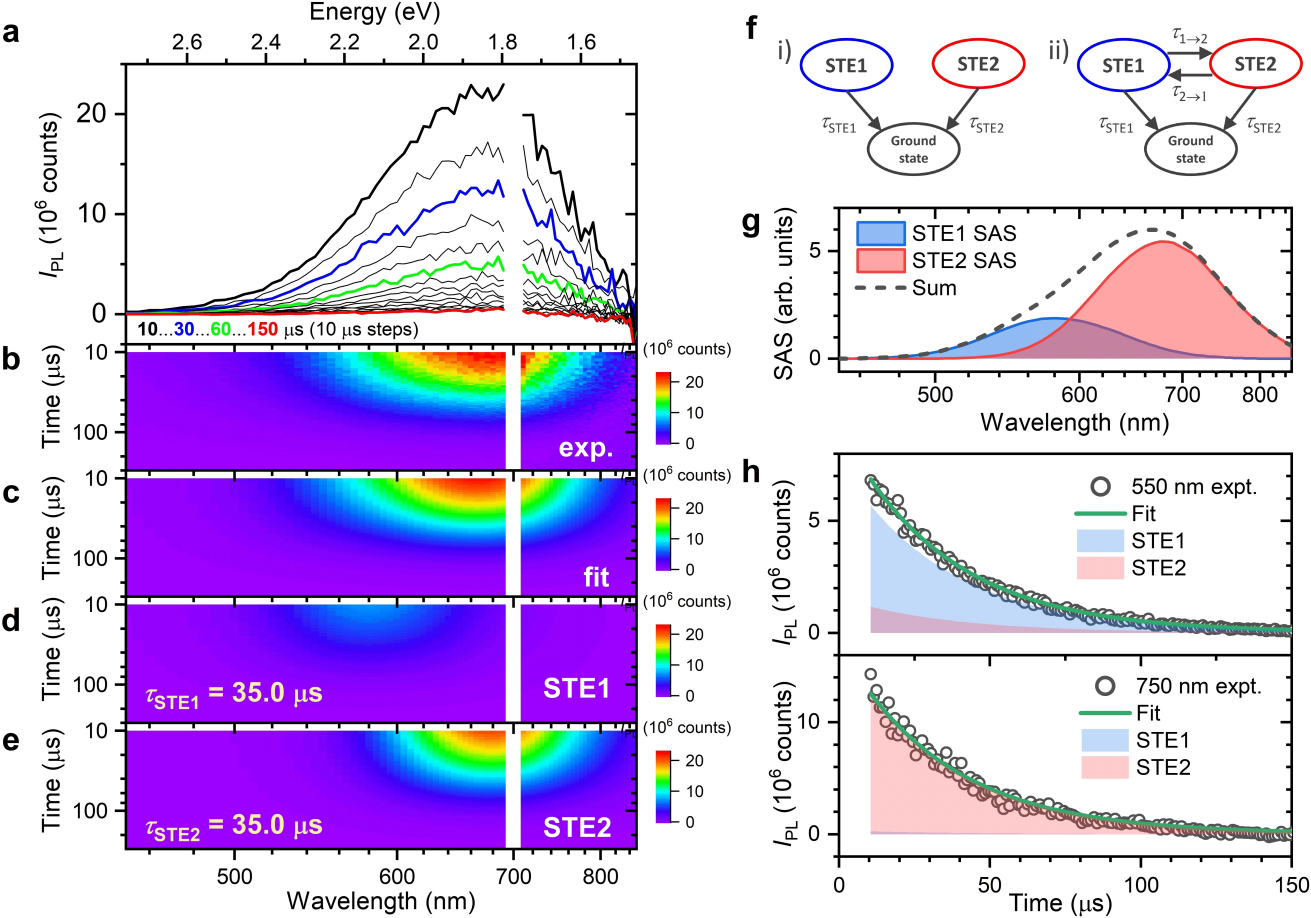
Analysis of time‐resolved broadband photoluminescence spectra of an [N(C_3_H_7_)_4_]_2_[Cu_4_Br_6_] thin film at 298 K upon pulsed photoexcitation at 350 nm. (a) Selected transient emission spectra in 10 μs time steps, with spectra at 10, 30, 60 and 150 μs highlighted by black, blue, green and red thick lines, respectively. (b) Contour plot of the same experimental data as in panel a. (c)–(e): Contour plot of the result of the global kinetic analysis for the overall fit, the contribution of STE1 only, and the contribution of STE2 only, respectively. (f) Model applied for the kinetic analysis of the transient emission spectra (i) and an alternative model involving a fast equilibrium between STE1 and STE2 (ii). (g) Resulting species‐associated spectra (SAS) for STE1 (blue) and STE2 (red) used in the global kinetic analysis, and their sum (black dashed line). (h) Two examples of kinetic traces (open circles) for the emission at 550 nm (top) and 750 nm (bottom). The fits obtained from the global kinetic analysis are shown as green solid lines, with the individual contributions of STE1 and STE2 indicated by the blue‐ and red‐filled areas, respectively.

In this section, we would like to discuss whether the two self‐trapped exciton states STE1 and STE2 are in a fast equilibrium or relax independently of each other. We compared first the PL lifetimes of the two species using transient broadband photoluminescence experiments at 298 K. Selected time‐resolved PL spectra of an [N(C_3_H_7_)_4_]_2_[Cu_4_Br_6_] thin film on quartz are shown in Figure 5a for the time range 10.5–150 μs upon photoexcitation at 350 nm. The gap in the spectrum arises from second‐order contributions of the excitation light and is therefore omitted. Figure 5b shows a two‐dimensional contour map of the PL decay for delay times of up to 150 μs and an emission wavelength range of 450–850 nm.

We performed a global kinetic analysis to fit the transient emission spectra, as summarized in Figure 5c–e. The kinetic model used here is based on the monoexponential decay of two independent species STE1 and STE2, each of which has a Gaussian band shape and decays with its own individual time constant (*τ*_STE1_ and *τ*_STE2_, see Figure 5f, model i, left side). The results of the global kinetic analysis are shown in Figure 5c for the overall fit, and the individual contributions of the species STE1 and STE2 are provided in Figure 5d and e, respectively. During the global kinetic analysis, the species‐associated spectra (SAS) of STE1 and STE2 were optimized to fit the experimental data.

The resulting spectra of STE1 and STE2 are shown in Figure 5g and are consistent with the spectral analysis performed in Figure 4 for the temperature of 298 K. We note that during the global kinetic analysis, the relative amplitudes for the SAS of STE1 and STE2 were fixed at the value obtained from the analysis of the PL spectrum at 298 K in Figure 4.

Figure 5h shows two examples of emission decays at 550 nm and 750 nm. The experimental time traces were well reproduced by the fit from the global kinetic analysis. The individual contributions of STE1 and STE2 are shown for each probe wavelength, and the fitted decay time constant *τ*_STE1_ (=*τ*_STE2_) was 35 μs. Such a long lifetime is consistent with the phosphorescence of a triplet state.

Given the similarity of the time constants *τ*_STE1_ and *τ*_STE2_ at 298 K, this measurement alone cannot determine whether STE1 and STE2 relax independently (Figure 5f, model i, left side) or are in a rapid equilibrium with the additional time constants *τ*_1→2_ and *τ*_2→1_, where *τ*_1→2_, *τ*_2→1_≪*τ*_STE1_, *τ*_STE2_ (Figure 5f, model ii, right side). In the first case, the experimentally measured rate constant *k*_exp_ at both wavelengths would be accidentally the same, i. e. *k*_exp_=*k*_STE1_≈*k*_STE2_ (*τ*_STE1_≈*τ*_STE2_). In the second case, after fast equilibration, the measured rate constant *k*_exp_ of the slow decay would be always the same at both wavelengths and depend on the specific values of the individual rate constants *k*_STE1_ and *k*_STE2_ of the two STE states. To distinguish between these two scenarios, further kinetic analyses over a wide temperature range are therefore desirable, as described in the following section.

### Temperature‐Dependent TCSPC Experiments

**Figure 6:**
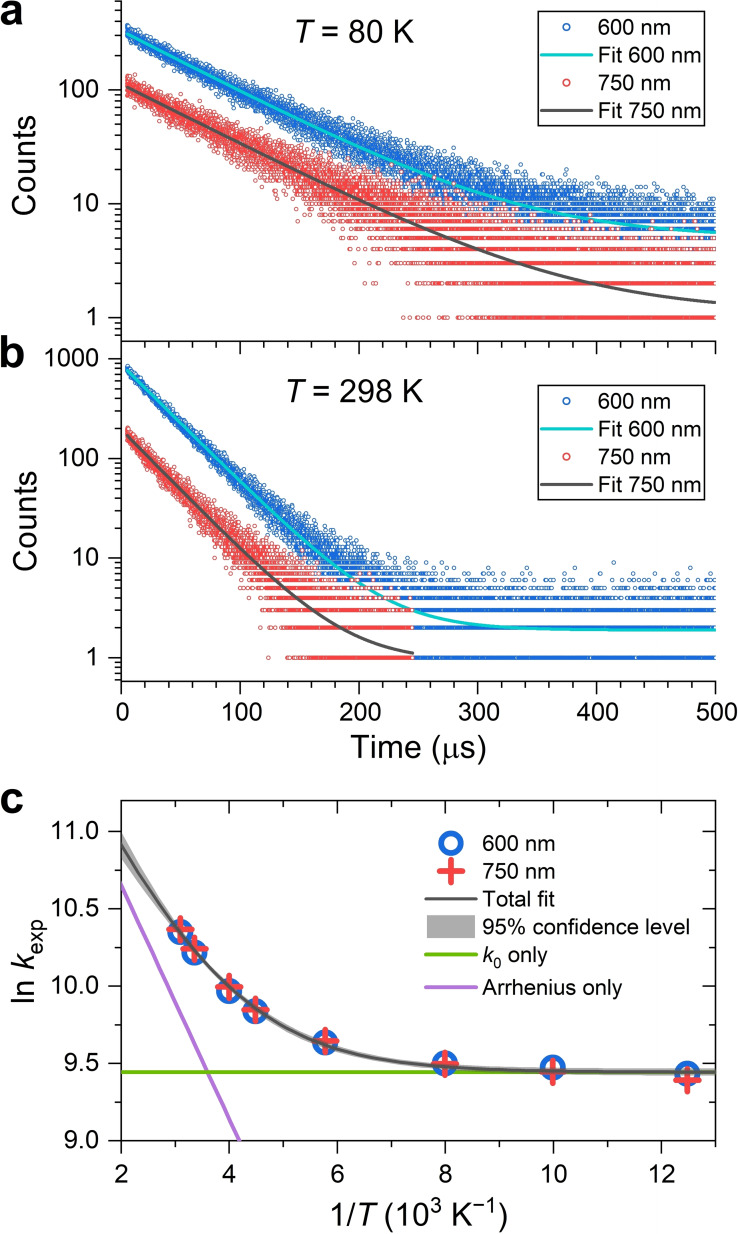
Temperature‐dependent TCSPC data of an [N(C_3_H_7_)_4_]_2_[Cu_4_Br_6_] thin film on quartz after photoexcitation with bursts of LED pulses at an excitation wavelength of 273 nm. (a) PL decays at *T*=80 K measured for two emission regions: (600±20) nm (blue symbols, predominantly STE1) and (750±20) nm (red symbols, predominantly STE2). Monoexponential fits with a constant offset are shown as cyan and black solid lines, respectively. (b) Same as panel a, but at *T*=298 K. (c) Arrhenius plot over the temperature range 80–323 K of the experimentally measured rate constants *k*_exp_ including the fit (black solid line) based on eq. (4) with the 95 % confidence interval shown as a grey‐shaded area. The green and violet solid lines show the contributions of the constant term (*k*_0_) and the Arrhenius term, respectively.

We investigated the temperature‐dependent PL decays of the STE1 and STE2 species using time‐correlated single photon counting (TCSPC). Figure 6a,b shows representative emission decays of an [N(C_3_H_7_)_4_]_2_[Cu_4_Br_6_] thin film on quartz after photoexcitation by bursts of LED pulses at 273 nm at the temperatures 80 K and 298 K, respectively. By using different bandpass filters, we focused on the two spectral regions of (600 ± 20) nm and (750 ± 20) nm, in which either the emission of the STE1 band or that of the STE2 band dominates (see Figure 4b). Table 1 summarizes the lifetimes and their error limits for these two probe wavelength ranges for the different temperatures.

**Table 1:** Summary of lifetimes *τ*_exp_ from the TCSPC experiments for an [N(C_3_H_7_)_4_]_2_[Cu_4_Br_6_] thin film over the temperature range 80–323 K including error limits Δ*τ*_exp_ (95 % confidence interval) for an excitation wavelength *λ*_pump_=273 nm.

*T* (K)	*τ*_exp_±Δ*τ*_exp_ (μs)	
	*λ*_probe_=(600±20) nm	*λ*_probe_=(750±20) nm
80	80.0±0.5	83.4±1.0
100	76.9±0.5	79.0±0.9
125	74.9±0.5	75.0±0.8
173	65.3±0.5	64.7±1.1
223	53.4±0.3	52.8±0.5
250	46.9±0.2	45.6±0.5
298	36.5±0.1	35.6±0.4
323	32.0±0.1	31.4±0.4

The kinetic decays can be well described for both wavelength ranges at all temperatures by a monoexponential function with constant offset (the latter results from the dark counts of the detector). It is important that at any given temperature the time constants in the different emission wavelength ranges are practically identical. The measured time constants vary by up to a factor 2.7 over the temperature range investigated, and it is unlikely that independently decaying STE1 and STE2 species exhibit exactly the same temperature dependence. We therefore assume that STE1 and STE2 are in a rapid equilibrium and therefore model ii) in Figure 4f is more consistent with the experimental observation. This means that even at 80 K, where the average thermal energy *k*_B_ *T* is 6.9 meV, any barrier for the interconversion between STE1 and STE2 (Δ*E*_1_ and Δ*E*_2_ in Figure 4g) must be small, on the order of a few meV or less. Therefore, the rate constants for the forward and reverse reactions in the equilibrium should be considerably larger than the rate constants for phosphorescence from STE1 and STE2.

The temperature‐dependence of the experimental rate constants *k*_exp_=*τ*_exp_^−1^ over the range 80–323 K was further analyzed based on the Arrhenius‐type plot (ln *k*_exp_ vs. 1/*T*) shown in Figure 6c. While the rate constant *k*_exp_ is almost temperature‐independent at low temperatures, it increases significantly at higher temperatures. The latter observation would be compatible with an Arrhenius‐type process. Consequently, we fitted the data points with the following expression:

(4) $$k_{\exp}\left(T\right)=k_{0}+k\left(T\right)=k_{0}+A\;\exp\left(-\frac{E_{a}}{k_{B}T}\right)$$

Here, *k*_0_ is the temperature‐independent part of the rate constant, and *k*(*T*) is an additional temperature‐dependent term, where *A* is the Arrhenius prefactor and *E*_a_ is the Arrhenius activation energy. The fit provides values of *k*_0_=(1.26±0.01)×10^4^ s^−1^, corresponding to a lifetime *τ*_0_ (=*k*_0_^−1^) of (79.2±0.6) μs, *E*_a_=(65±3) meV and *A*=(1.9±0.2)×10^5^ s^−1^.

In the following, we consider possible interpretations of the experimentally determined temperature dependence. Very often, the observed behavior in semiconductor and molecular systems is interpreted as the sum of a temperature‐independent or only weakly dependent process (mainly due to radiative decay, associated with the rate constant *k*_0_=*k*_rad_ in eq. (4)) combined with a temperature‐dependent nonradiative process (rate constant *k*(*T*)=*k*_nonrad_(*T*) in eq. (4)).[^31^, ^33^, ^37^, ^38^, ^39^, ^40^, ^41^] However, such a mechanism cannot be used here for the following reason. With our fit parameters we can estimate the expected temperature‐dependent PL quantum yield *Φ*_rad_(*T*) for our case via

(5) $$\Phi_{\mathrm{rad}}\left(T\right)=\frac{k_{\mathrm{rad}}}{k_{\mathrm{rad}}+k_{\mathrm{nonrad}}\left(T\right)}$$

For *k*_rad_, we use the above‐mentioned fitting value of 1.26×10^4^ s^−1^ and for *k*_nonrad_(*T*) the corresponding Arrhenius expression (*E*_a_=65 meV, *A*=1.9×10^5^ s^−1^). This would give a *Φ*_rad_ value of 100 % at 80 K (essentially only radiative decay) and about 45 % at 298 K, which would demonstrate competition from the nonradiative channel. However, previous measurements of the absolute PL quantum yield of [N(C_3_H_7_)_4_]_2_[Cu_4_Br_6_] at 298 K by Tian et al. and Chen et al. showed a radiative quantum yield in the range 95–97 %.[^4^, ^12^] Therefore, such a thermally activated nonradiative process cannot occur.

In fact, what we observe here for [N(C_3_H_7_)_4_]_2_[Cu_4_Br_6_] is a rather rare example of “negative thermal quenching (NTQ)” of photoluminescence,[^32^, ^42^, ^43^, ^44^] which leads to a brightening of the photoluminescence with increasing temperature (compare Figure 4f). One possibility could be that the radiative rate constant increases with temperature more than the rate constants of the competing nonradiative processes. Alternatively, detrapping from shallow defect states, which are present in this compound (see the Urbach tail in Figure 3d), might increase the PL quantum yield at higher temperature.^[43]^

The pure NTQ behavior of [N(C_3_H_7_)_4_]_2_[Cu_4_Br_6_] observed in this temperature range differs considerably from previous results for other halocuprates(I). For instance, in the case of the compound [N(C_4_H_9_)_4_][CuBr_2_], Peng et al. found an increase of the PL intensity in the range 80–158 K, followed by a decay up to 298 K.^[45]^ For [N(C_2_H_5_)_4_]_2_[Cu_2_Br_4_], Liu et al. observed “normal” thermal quenching behavior, with a substantial reduction of the PL quantum yield in the temperature range 77–300 K accompanied by a slight increase of the PL lifetime from 36 to 52 μs.^[6]^ Considering non‐copper(I) systems, for the 1D hybrid organic‐inorganic tin halide [ODA][Sn_2_I_6_] (ODA=1,8‐octyldiammonium), Pullerits, Chen and co‐workers found “normal” thermal quenching behavior, with a substantial decrease of the PL quantum yield and lifetime with increasing temperature. The behavior was assigned to an increase of the nonradiative rate constant (accompanied by a decrease of the radiative rate constant), resulting in an Arrhenius‐type behavior with an apparent activation energy of 466 meV.^[33]^ As a final example, for high‐quality samples of the organic–inorganic tin perovskite [FA][SnI_3_] (FA=formamidinium), Kahmann et al. observed a complicated temperature dependence, with a decrease of the PL intensity over the range 4–130 K, an intermediate regime of NTQ behavior (130–185 K) and then again a decrease of the PL intensity up to 298 K.^[32]^ In that case, the NTQ behavior was not observed for “low‐quality” samples, suggesting that NTQ appears only for samples in which nonradiative electron–hole recombination is efficiently suppressed.^[32]^

### Broadband Transient Absorption Experiments

**Figure 7:**
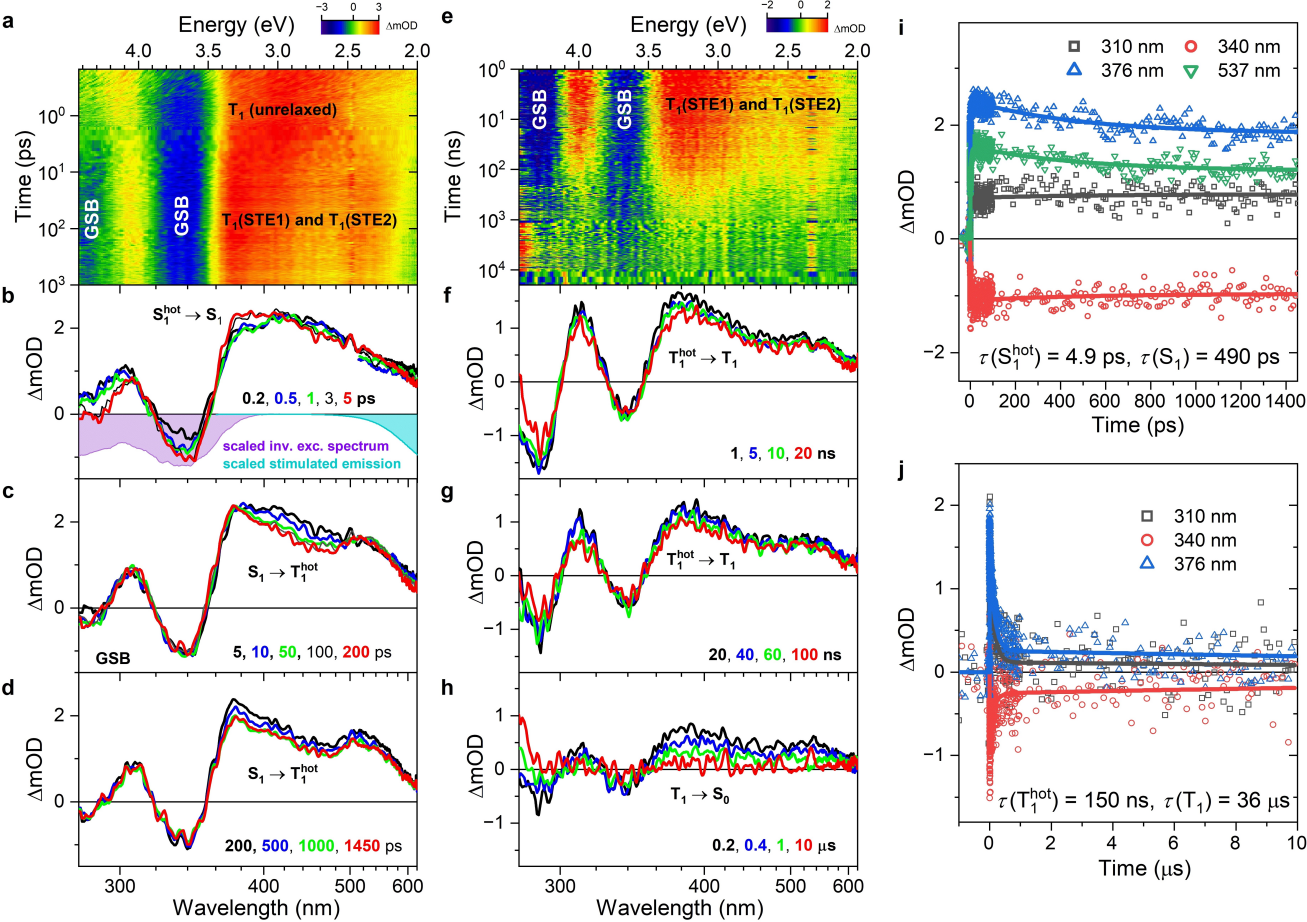
Broadband transient absorption spectroscopy of an [N(C_3_H_7_)_4_]_2_[Cu_4_Br_6_] thin film at 298 K. (a) Transient absorption spectra after photoexcitation by femtosecond pulses at 250 nm (pulse length 80 fs) shown as a contour plot. Note the logarithmic time axis. (b)–(d) Transient absorption spectra at selected pump–probe delay times up to 1450 ps, with assignments of kinetic processes between the different electronic states involved. The violet‐colored area and the cyan‐colored area are the scaled and inverted steady‐state excitation spectrum and the scaled steady‐state stimulated emission spectrum, respectively. (e)–(h) Same as in panels (a)–(d), but for time scales up to 10 μs upon excitation with subnanosecond pulses at 266 nm (pulse length 420 ps). (i) Kinetic traces up to 1450 ps at the four wavelengths indicated (colored symbols) including fits from the global analysis (colored lines) using the kinetic model described in the text. Time constants obtained from the fit are included. (j) Same as in panel i, but for longer time scales up to 10 μs.

Further information regarding the relaxation dynamics of [N(C_3_H_7_)_4_]_2_[Cu_4_Br_6_] can be obtained from UV–Vis broadband transient absorption experiments covering the femtosecond to microsecond time scale. Figure 7a contains a contour plot of the transient spectra up to 1000 ps for excitation with femtosecond laser pulses at 250 nm, and panels b–d show selected transient absorption spectra up to a time delay of 1450 ps. Around a time delay of zero (Figure 7b), negative spectral bands appear at 285 and 340 nm, which are located at the same position as the peaks of the inverted steady‐state excitation spectrum (violet area). Using the terminology employed for molecular electronic states, which is justified by the molecular salt structure of these closed‐shell compounds, these bands can be assigned to a ground‐state bleach (GSB) contribution of the singlet state S_0_. In addition, we observe overlapping broad excited‐state absorption (ESA) bands in the wavelength ranges 290–320 nm and 350–620 nm. As we deal with very short times (200 fs) after photoexcitation, these ESA bands are assigned to the hot singlet state S_1_^hot^ (or in band‐model terminology a localized hot exciton state LE^hot^). In the time range 0.2–5 ps, we observe changes in the ESA bands, with an increase around 380 nm and decreasing amplitudes at about 295 and 460 nm. At the same time, the GSB feature at 340 nm becomes more negative. This spectral evolution is consistent with a narrowing of the ESA bands, which can be associated with a vibrational cooling in the excited state (S_1_^hot^→S_1_).

On time scales up to 1450 ps (Figure 7c,d), we observe a pronounced decay of the ESA band in the range 380–490 nm, however in this case without any spectral changes in the region below 380 nm, except for minor variations around 285 nm which arise from residual stray light from the spectral wing of the 250 nm pump beam. We identify these dynamics with the intersystem crossing (ISC) process S_1_→T_1_^hot^. The assignment to a triplet state is consistent with the fact that we do not see indications for a negative stimulated emission (SE) band. Note that an SE band for an allowed transition would appear above 480 nm (compare the cyan‐shaded steady‐state SE spectrum in Figure 7b). However, because of the optically forbidden nature of the T_1_→S_0_ transition, its oscillator strength transition is very weak and not detectable in the transient absorption signal on top of the strongly allowed ESA transitions. This assignment to a triplet state is also consistent with the long lifetime of the emissive state obtained in the transient PL experiments (Figures 5 and 6).

Figure 7e–h displays the corresponding transient absorption data covering the time scale up to 10 μs for excitation by subnanosecond pulses at 266 nm. The spectral evolution of the ESA bands in Figure 7f,g, which does not show a decay in the GSB region around 340 nm, is assigned to an excited‐state process, in this case cooling of the triplet state (T_1_^hot^→T_1_). We note that the ESA band of the triplet state shows two maxima at 390 and 530 nm, which can be tentatively assigned to ESA of the two STE states to higher excited triplet states T_n_, i. e. T_1_(STE1)→T_n_(STE1) and T_1_(STE2)→T_n_(STE2). On even longer time scales (Figure 7h), we see a decrease of all bands, which is associated with the decay from T_1_ to the ground electronic state S_0_.

Representative kinetics of [N(C_3_H_7_)_4_]_2_[Cu_4_Br_6_] at selected probe wavelengths are provided in panels i and j of Figure 7 for delay times of up to 1450 ps and 10 μs, respectively. The relaxation processes were modeled using a global analysis based on the sequential kinetic scheme6

(6)

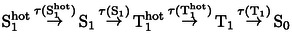




**Figure 8:**
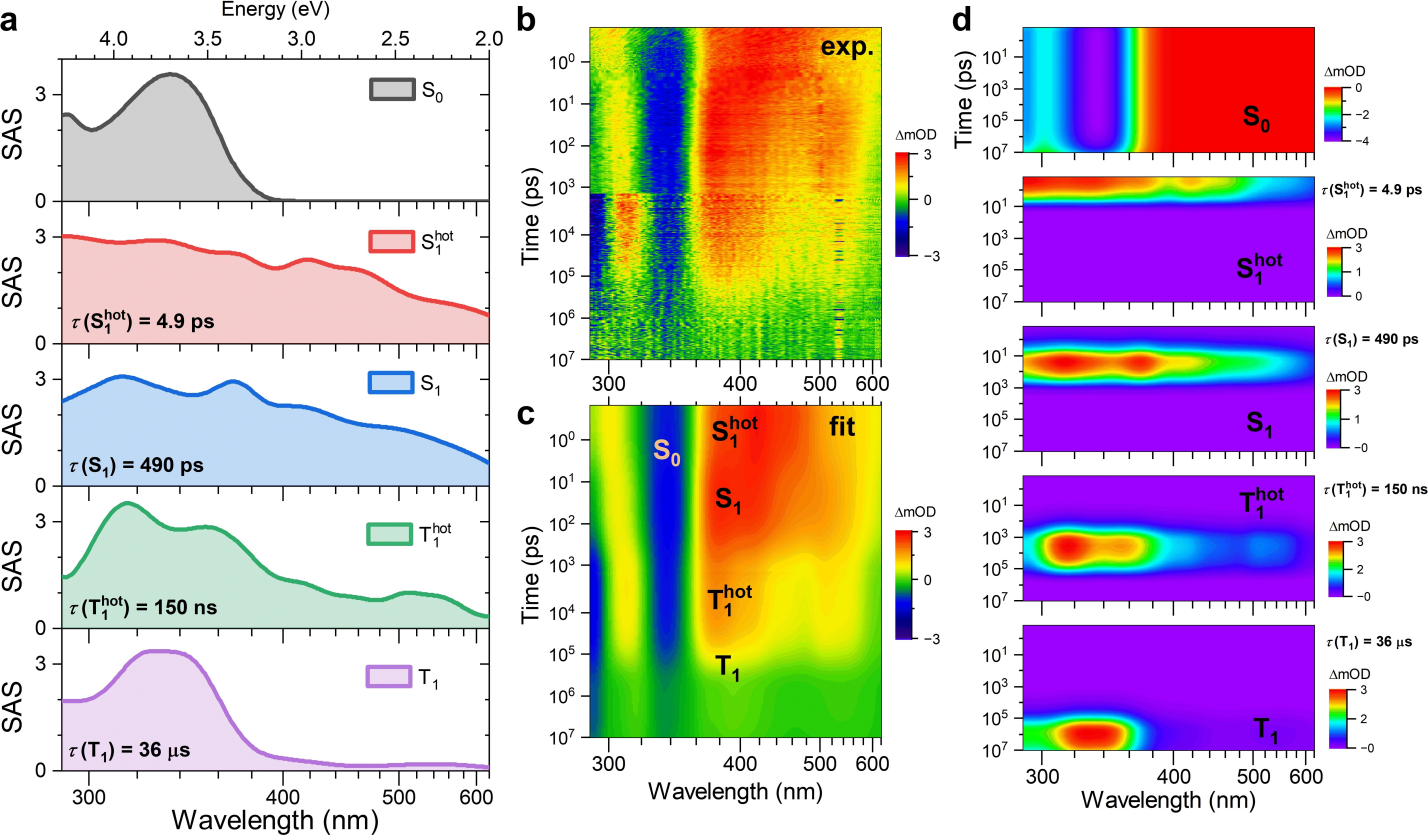
Results from the global kinetic analysis of the broadband transient absorption spectra of [N(C_3_H_7_)_4_]_2_[Cu_4_Br_6_]. (a) Species‐associated spectra (SAS) for the species S_0_, S_1_^hot^, S_1_, T_1_^hot^ and T_1_, with associated time constants for the excited‐state species. The S_0_ spectrum represents a fit to the steady‐state excitation spectrum. (b) Combined contour plot of the experimental transient absorption spectra from the subpicosecond range up to 10 μs. Note the logarithmic time axis. (c) Corresponding contour plot obtained from the best fit, as extracted from the global kinetic analysis of the experimental data shown in panel b. (d) Contour plots showing the individual contributions of the different species to the contour plot in panel c.

This analysis provided the species‐associated spectra and their corresponding time constants *τ* (=*k* ^−1^). The results are summarized in Figure 8. The SAS of the hot S_1_ state is broad and spans the entire spectral range investigated (Figure 8a). The fitted lifetime *τ*(S_1_^hot^) (=*k*(S_1_^hot^)^−1^) is 4.9 ps. This time scale is typical for the vibrational relaxation of molecular species in condensed‐phase environments.[^7^, ^9^, ^46^, ^47^, ^48^] The relaxed S_1_ species has a slightly narrower and more structured spectrum than S_1_^hot^ (Figure 8a), and its lifetime *τ*(S_1_) (=*k*(S_1_)^−1^) is 490 ps. This decay time constant can be compared with ISC time constants obtained for other bromocuprates(I) having a 0D molecular‐salt structure, such as [CH_3_NH_3_]_4_[Cu_2_Br_6_] (61 ps)^[9]^ and [N(C_2_H_5_)_4_]_2_[Cu_2_Br_4_] (184 ps).^[7]^ The intermediately formed hot triplet state T_1_^hot^, has a distinctly different SAS (Figure 8a) and an associated time constant *τ*(T_1_^hot^) (=*k*(T_1_^hot^)^−1^) of 150 ns. This value is compatible with slow cooling by heat transfer in the film via acoustic phonon relaxation, again in reasonable agreement with previous results obtained for other bromocuprates(I).[^7^, ^9^] In the last step, the relaxed T_1_ state decays to S_0_ by phosphorescence with the time constant *τ*(T_1_) (=*k*(T_1_)^−1^) of 36 μs. The latter value was taken from the TCSPC experiments (Table 1), because of their better signal‐to‐noise ratio.

The best fit obtained from the global kinetic analysis matches the experimental data very well. This is demonstrated by the comparison of the experimental and simulated contour plots in Figure 8b,c. Individual contributions to the contour plot for each species are summarized in Figure 8d. We note that it is not possible to unambiguously assign individual spectral features to the STE states T_1_(STE1) and T_1_(STE2), because their lifetimes are practically identical (Table 1).

We also carried out additional transient absorption experiments, in which the pump fluence was varied systematically. The fluence‐dependent kinetics showed no obvious changes in their shape (Supporting Information, Supporting Note 2). This finding suggests that higher‐order processes, such as triplet–triplet annihilation (TTA), can be excluded at the pump beam fluences employed in the current experiments.

## Conclusions

The efficient dual emission of the bromocuprate(I) compound [N(C_3_H_7_)_4_]_2_[Cu_4_Br_6_] arises from two triplet‐state structures of the [Cu_4_Br_6_]^2−^ anion which are in an equilibrium. The time constants for the forward and backward reaction in this equilibrium must be considerably shorter than the triplet lifetime, because the time constants measured in both emission bands are practically identical. These triplet structures, which are populated by intersystem crossing from the initially populated singlet state on a 500 ps time scale, are further stabilized by relaxation of the surrounding lattice. This relaxation results in the formation of two distinct self‐trapped exciton states, denoted as STE1 and STE2, and these states are characterized by very large Huang‐Rhys factors. Moreover, the compound displays negative thermal quenching over the temperature range 80–323 K, with a decrease of the lifetime from 82 to 32 μs. This is a rather rare, advantageous effect for such emitters, which otherwise often experience unfavorable, increasing competition from nonradiative processes and thus a quenching of the photoluminescence.

The high PL quantum yield of more than 95 % at room temperature is encouraging for lighting applications, as is the lifetime of several tens of microseconds. Therefore, such a halocuprate(I) compound or mixtures of such compounds showing multiple STE bands might be a good choice for applications requiring panchromatic whitelight emission.[^49^, ^50^] On the other hand, the intrinsically broad emission of STE states, originating from an excited emissive state, which is far away from the ground state equilibrium configuration, comes at the cost of color purity. For instance, at 298 K, the fitted FWHM of the STE1 and STE2 emission bands is 421 and 433 meV, respectively, and for the previously investigated [CH_3_NH_3_]_4_[Cu_2_Br_6_] (0D) and the all‐inorganic Cs[Cu_2_I_3_] (1D) it is 480 and 410 meV, respectively.[^9^, ^51^] In contrast, for the prototypical organic–inorganic lead perovskite material [CH_3_NH_3_][PbI_3_] a much smaller value of 93 meV was determined.^[52]^ It remains to be seen, how other members of the organic–inorganic halocuprate(I) family, based on either 0D, 1D or 2D structural motifs perform in this respect and how their photophysical properties can be tuned further by deliberate variation of the countercation. Investigations along these lines are currently ongoing in our laboratory.

## Experimental Section

### Thin‐Film Preparation

CuBr (Sigma‐Aldrich, 99.999 %) and [N(C_3_H_7_)_4_]Br (Sigma‐Aldrich, 98 %) were dissolved in a 2 : 1 molar ratio in DMSO (Acros Organics, 99.7 %, extra dry) at a nominal composition of 20–25 wt % [N(C_3_H_7_)_4_]_2_[Cu_4_Br_6_]. To prevent oxidation of copper(I) to copper(II), 1 μL of hypophosphorous acid (Alfa Aesar, 50 wt %) was added to the solution. Thin films were then deposited by spin‐coating the solution at 500 rpm for 4 s and then 2000 rpm for 30 s onto pre‐heated quartz (Tempotec Optics Co., Ltd.) or borosilicate glass substrates, which were thoroughly cleaned and irradiated by UV−C light beforehand. Immediately after spin‐coating, the thin films were post‐annealed at 80 °C for 30 min to remove residual solvent. A protective layer was spin‐coated on top of the film (500 rpm for 4 s followed by 2000 rpm for 30 s) using a 12.5 mg mL^−1^ solution of poly(methyl methacrylate) (PMMA, Alfa Aesar) in anhydrous chlorobenzene (Sigma‐Aldrich, 99.8 %). This way, the film was protected against the influence of humidity and oxygen during some measurements under ambient atmosphere. After the second spin‐coating step, the samples were annealed again at 80 °C for 30 min to remove residual chlorobenzene.

### X‐Ray Diffraction and Scanning Electron Microscopy

Thin‐film X‐ray diffractograms (Figure 1) were measured on a PANalytical X'Pert MPD PRO diffractometer using copper radiation (Kα_1_=1.54060 Å, Kα_2_=1.54443 Å). A Rietveld refinement was employed to analyze the X‐ray diffractograms using the program MAUD considering also texture effects of the thin film.^[17]^ The structure was visualized using the program VESTA.^[18]^ Scanning electron microscopy images (Figure 2) were obtained on an FEI Quanta 250 FEG instrument.

### Transmission and Photoluminescence Microscopy

Transmission and photoluminescence images (Figure 2) were recorded using an inverted microscope (Olympus IX71) equipped with 40x and 4x objectives (Olympus LCPLFL, NA 0.60, and UPLFL, NA 0.13) combined with a thermoelectrically cooled CCD camera (PCO Sensicam QE). In the PL microscopy experiments, the sample was illuminated by light at 330 nm with an FWHM of 14 nm obtained from a 150 W xenon lamp / monochromator combination (Till Photonics Polychrome 5000) which was coupled into a quartz fiber and then directed through the microscope objective. The PL emitted by the sample was sent through a longpass filter (440 nm) to remove any residual excitation light. Images were recorded using the TILL Photonics Live Acquisition Software.

### Steady‐State Absorption and Photoluminescence

Steady‐state absorption spectra of the thin films at 298 K (Figure 3) were measured on a Varian Cary 5000 spectrophotometer with a slit width and step size of 0.5 nm. Steady‐state PL experiments were carried out at 298 K using an Agilent Cary Eclipse spectrophotometer with the excitation and emission slit widths set at 5 nm (Figure 3).

### Transient Broadband Photoluminescence

Photoluminescence kinetics were measured on an Agilent Cary Eclipse spectrophotometer. The excitation light at 350 nm was obtained from a pulsed xenon lamp (80 Hz, FWHM 2 μs) combined with a monochromator. The PL was detected over the wavelength range 450–850 nm every 5 nm with the exception of the wavelength range 695–705 nm. The latter region was excluded because of stray‐light signals arising from second‐order contributions of the pump light in the emission monochromator. Individual kinetics were collected with a gate time of 5 μs and a time step of 1 μs up to a total decay time of 1 ms. The recording started after a delay time of 10 μs to exclude any contributions by the trailing edge of the lamp pulse. Ten of these kinetic traces were averaged for each wavelength. Transposition of the wavelength‐dependent kinetics provided a set of time‐dependent PL spectra, which were afterwards corrected for the wavelength‐dependent sensitivity of the emission monochromator / detector combination (Figure 5).

### Temperature‐Dependent Photoluminescence

PL spectra and kinetics over the temperature range 80–323 K were recorded using a newly established setup based on a customized temperature‐regulated stage (Linkam HFSX350‐CAP) combined with a liquid‐nitrogen cooling system (Linkam LNP96) and a temperature controller (Linkam T96). The thin‐film samples were mounted inside the stage on a nickel‐plated copper block which was connected with the liquid‐nitrogen cooling system and contained heater elements as well as a Pt100 resistance temperature detector. The block had an optical aperture of 4.5 mm. Two ports with fused silica windows (Spectrosil 2000) sealed by silicone rubber rings on the top and bottom of the stage provided optical access for the spectroscopic experiments. The outer cell body was kept at a temperature of 50 °C by a thermostat (Lauda RE 106), and the windows were constantly flushed from the outside with dry nitrogen to avoid any condensation of ambient humidity during the experiments.

For steady‐state PL measurements (Figure 4), the stage was interfaced with an optical setup mounted inside a light‐tight SM1 cage system (Thorlabs). The emission of a UV‐LED (Thorlabs M365LP1, 365 nm, FWHM 10 nm) was passed through a filter (Schott UG1) and collimated by a quartz lens. The light was then reflected off a longpass dichroic mirror (Thorlabs DMLP425R, 425 nm cut‐on wavelength) at a 90° angle and focused onto the sample by a quartz lens. The resulting photoluminescence traveled back through the longpass dichroic mirror and was reflected off an aluminum mirror at a 90° angle. The PL was collimated by a quartz lens and was then focused into a 600 μm diameter optical fiber (Avantes FC‐UVIR600‐1‐BX), which was connected to a spectrograph equipped with a back‐illuminated, thermoelectrically‐cooled CCD detector (Avantes AvaSpec‐HERO).

For the TCSPC experiments (Figure 6), a similar setup based on a light‐tight SM1 cage system was employed. Here, the output of a pulsed LED (Becker & Hickl UVL‐FB‐270) with a center wavelength of 273 nm was collimated by a quartz lens and sent through a wire‐grid polarizer (Thorlabs WP25M‐UB) set at vertical polarization (0°) and a filter (Schott UG11). The light was then reflected off a longpass dichroic mirror (AHF HCR325, 325 nm cut‐on wavelength) at a 90° angle and focused onto the sample by a quartz lens. The photoluminescence then traversed the longpass dichroic mirror and was reflected off an aluminum mirror at a 90° angle. It was collimated by a lens and then passed another wire‐grid polarizer (Thorlabs WP25M‐UB) which was set at the magic angle with respect to the initial polarization of the LED light. A well‐defined spectral range of the photoluminescence was selected by a bandpass filter (Thorlabs FBH600‐40 or FBH750‐40) and then refocused by another quartz lens onto a hybrid multialkali photodetector (Becker & Hickl, HPM‐100‐07). The signal pulses from the detector and the synchronization pulses of the UV‐LED were fed into the inputs of a TCSPC module (Becker & Hickl, SPC‐130IN). To detect the slow decay of the photoluminescence, the TCSPC module was operated in its triggered‐accumulation multichannel scaler (MCS) mode using a Stanford Research Systems DG535 digital delay / pulse generator. Here, 2 μs long bursts from the 80 MHz LED pulse train (160 pulses in total) were employed at a repetition frequency of 1 kHz. Additional details of the TCSPC setup can be found in our previous publications.[^7^, ^53^] The analysis of the PL decays was performed by the program FAST (Edinburgh Instruments). The time constants were obtained from tail fits using a monoexponential decay plus a constant offset. A support‐plane analysis was applied to extract the error limits of the time constants for a confidence interval of 95 %.

### Broadband UV–Vis Transient Absorption

Ultrafast broadband transient absorption experiments in the UV–Vis region (260–700 nm) up to a pump–probe delay time of 1500 ps were performed at 920 Hz repetition frequency using a setup described previously, which has a time resolution of 80 fs^[54]^ and utilizes the pump–supercontinuum probe (PSCP) approach.^[55]^ The thin films were excited using the 250 nm output of an OPA system (Coherent OPerA Solo) which was mechanically chopped at 460 Hz. For the measurements on time scales up to 10 μs, the fourth harmonic of a Q‐switched Nd:YAG microlaser (Standa Q1TH)^[9]^ was used for exciting the [N(C_3_H_7_)_4_]_2_[Cu_4_Br_6_] thin film. The sample was placed inside a home‐built aluminum cell which was constantly flushed by dry nitrogen. The cell was mounted on an *x*–*y* piezo stage and moved within an area of 2×2 mm^2^.

The transient spectra (Figure 7) were subjected to a global kinetic analysis procedure[^56^, ^57^] which employed the kinetic scheme of eq. (6) (Figure 8). The steady‐state thin‐film excitation spectrum was kept fixed and the species‐associated spectra and time constants of the excited‐state species were optimized during the fitting procedure.

## Conflict of Interests

The authors declare no conflict of interest.

1

## Supporting information

As a service to our authors and readers, this journal provides supporting information supplied by the authors. Such materials are peer reviewed and may be re‐organized for online delivery, but are not copy‐edited or typeset. Technical support issues arising from supporting information (other than missing files) should be addressed to the authors.

Supporting Information

## Data Availability

The data that support the findings of this study are available from the corresponding authors upon reasonable request.
