# Supplementary material for: Photoinduced Dynamics of [N(C3H7)4]2[Cu4Br6] Thin Films with Dual Self‐Trapped Exciton Emission and Negative Thermal Quenching
Source: Chemphyschem. 2025 Mar 25;26(11):e202401143. doi: 10.1002/cphc.202401143 (PMC12132918; doi:10.1002/cphc.202401143)
Supplement: Supplementary file 1 — Supporting Information [file CPHC-26-e202401143-s001.pdf]

ChemPhysChem

Supporting Information

Photoinduced Dynamics of $[N(C_3H_7)_4]_2[Cu_4Br_6]$ Thin Films with Dual Self-Trapped Exciton Emission and Negative Thermal Quenching

Domenic Gust, Alexander Merker, Konstantin Moritz Knötig, Kawon Oum,* and Thomas Lenzer*

Supporting Information for

Photoinduced Dynamics of $[N(C_3H_7)_4]_2[Cu_4Br_6]$ Thin Films with Dual Self-Trapped Exciton Emission and Negative Thermal Quenching

Domenic Gust,[a] Alexander Merker,[a] Konstantin Moritz Knötig,[a] Kawon Oum,*[a] and Thomas Lenzer*[a]

[a] University of Siegen, Faculty IV: School of Science and Technology, Department Chemistry and Biology, Physical Chemistry 2, Adolf-Reichwein-Str. 2, 57068 Siegen, Germany
E-mail: oum@chemie.uni-siegen.de, lenzer@chemie.uni-siegen.de

Table of Contents

Supporting Note 1. Configuration-Coordinate Model S2
Supporting Note 2. Fluence-Dependent Transient Absorption Experiments S4
Supporting References S5

Supporting Note 1.
Configuration-Coordinate Model

In this section, we describe in more detail the configuration-coordinate approach[1] which forms the basis for interpreting the Stokes shift of the halocuprate(I) emission. The simplified schemes in Supporting Figure 1 summarize the relevant states involved (using molecular state designations) and their energetics.

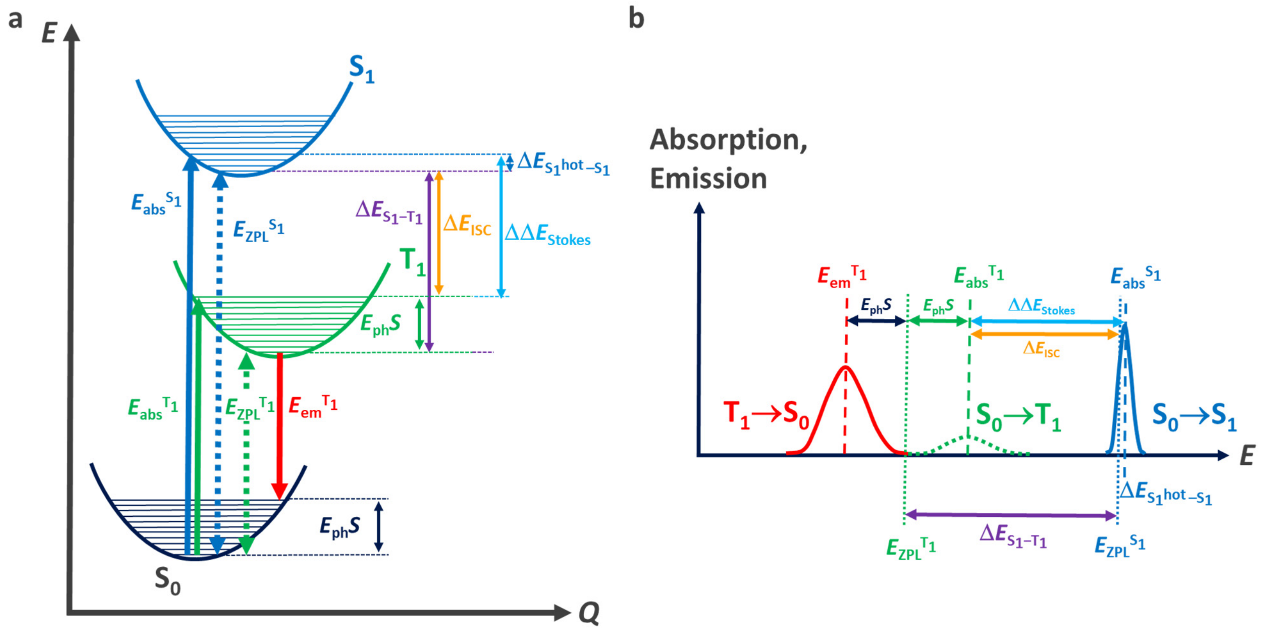

Supporting Figure 1. Schematic illustrations of the configuration-coordinate model. (a) Simplified configuration-coordinate diagram involving the potentials for the relevant halocuprate(I) anion states S_0, S_1 and T_1 (using molecular designations) with relevant energetic parameters indicated by arrows. The lines in each of the potential curves indicate different phonon levels. (b) Resulting schematic absorption and emission spectra with the same energetic parameters shown in panel a. Note that the $S_0 \rightarrow T_1$ transition (green dotted line) is optically forbidden assuming *L-S* coupling and therefore invisible in the experimental absorption spectrum.

The halocuprate(I) compound $[N(C_3H_7)_4]_2[Cu_4Br_6]$ investigated here exhibits phosphorescence. Therefore, we consider $S_0 \rightarrow T_1$ (absorption) and $T_1 \rightarrow S_0$ (emission). The configuration-coordinate model assumes that the Stokes shift arises from phonon relaxation in both states. The phosphorescence Stokes shift is defined as the difference between the peak of the $S_0 \rightarrow T_1$ absorption band and the peak of the $T_1 \rightarrow S_0$ emission band:

$$\Delta E_{\text{Stokes,Huang-Rhys}} = E_{\text{abs}}^{T_1} - E_{\text{em}}^{T_1} = 2\,E_{\text{ph}}S \qquad \text{(S1)}$$

In this simple model, an average phonon energy E_{ph} and an average Huang-Rhys factor S are assumed for S_0 and T_1, and in each state, an energy of $E_{ph}S$ is released to the lattice.

Now, the obvious difficulty is that the absorption transition $S_0 \rightarrow T_1$ is spin-forbidden (assuming *L–S* coupling). Consequently, it has a very small absorption coefficient and cannot be observed experimentally. What one can see in the absorption spectrum instead is the optically bright $S_0 \rightarrow S_1$

transition. Therefore, in the present case, experimentally we can only measure the Stokes shift of the $T_1 \rightarrow S_0$ emission band with respect to the $S_0 \rightarrow S_1$ absorption band, denoted below as $\Delta E_{Stokes,exp}$. This will be larger than the Stokes shift of the T_1 emission band with respect to the $S_0 \rightarrow T_1$ absorption band given by eq. (S1). As shown in Supporting Figure 1, the energy difference between the quantities is equal to the difference in the peak absorption energies of the S_1 and T_1 states:

$$\Delta\Delta E_{\text{Stokes}} = \Delta E_{\text{Stokes,exp}} - \Delta E_{\text{Stokes,Huang-Rhys}} = E_{\text{abs}}^{S_1} - E_{\text{abs}}^{T_1} = E_{\text{abs}}^{S_1} - E_{\text{em}}^{T_1} - 2\,E_{\text{ph}}S \qquad \text{(S2)}$$

At 298 K, we obtain $\Delta\Delta E_{Stokes}$ values of 0.26 eV and 0.67 eV for the triplet STE1 and STE2 states, respectively, using the emission peak positions from Figure 4d in the main manuscript. In general, there are two contributions to the energy difference $\Delta\Delta E_{Stokes}$. The first contribution is the excess energy $\Delta E_{S_1^{hot}-S_1}$ which is dissipated from the initially populated "hot phonon" S_1 singlet state as heat to the lattice, resulting in the formation a "cold phonon" S_1 state:

$$\Delta E_{S_1^{\text{hot}}-S_1} = E_{\text{abs}}^{S_1} - E_{\text{ZPL}}^{S_1} \qquad \text{(S3)}$$

Using $E_{abs}{}^{S_1}$ = 3.70 eV obtained from the peak of the experimental absorption spectrum (Figure 3b, main manuscript) and the energy of the zero-phonon line (ZPL) transition $E_{ZPL}{}^{S_1}$ = 3.30 eV (which corresponds to the direct band gap extracted from the Tauc plot in Figure 3c of the main manuscript) we arrive at a value for $\Delta E_{S_1^{hot}-S_1}$ of 0.40 eV. The second contribution is the excess energy released after isoenergetic ISC from S_1 to T_1 as heat to the lattice (omitting the energy portion $E_{ph}S$ in T_1 which is already considered in the coordination-coordinate model, see Supporting Figure 1):

$$\Delta E_{\text{ISC}} = \Delta\Delta E_{\text{Stokes}} - \Delta E_{S_1^{\text{hot}}-S_1} \qquad \text{(S4)}$$

The resulting values for ΔE_{ISC} are −0.145 eV and 0.268 eV for the triplet STE1 and STE2 states, respectively. Note that the negative value in the case of STE1 indicates that the zero-phonon level of S_1 lies 145 meV below the highest excited ($E_{ph}S$) phonon level of the T_1 state. Finally, the singlet–triplet energy gap $\Delta E_{S_1-T_1}$ defined as the difference of the zero-phonon line energies of the S_1 and T_1 states is given as:

$$\Delta E_{S_1-T_1} = E_{\text{ZPL}}^{S_1} - E_{\text{ZPL}}^{T_1} = \Delta E_{\text{ISC}} + E_{\text{ph}}S \qquad \text{(S5)}$$

This equation provides $\Delta E_{S_1-T_1}$ values of 0.51 eV and 0.87 eV for the triplet STE1 and STE2 states, respectively. The ZPL position of the T_1 state can be determined via

$$E_{\text{ZPL}}^{T_1} = E_{\text{em}}^{T_1} + E_{\text{ph}}S \qquad \text{(S6)}$$

resulting in $E_{ZPL}{}^{T_1}$ values of 2.79 eV and 2.43 eV for STE1 and STE2, respectively. We note that within the Huang-Rhys model the Stokes shift (2 $E_{ph}S$) arises from the displacement along the configuration coordinate Q of the two potentials involved in the transition. The model does not account for additional contributions to the Stokes shift, e.g. additional stabilization of the T_1 state by self-trapping. In fact, previous TDDFT calculations for the isolated $[Cu_2Br_6]^{4-}$ anion of the related halocuprate(I) system $[CH_3NH_3]_4[Cu_2Br_6]$, found that $\Delta E_{S_1-T_1}$ is only 0.34 eV.[2] Assuming the same energy difference $\Delta E_{S_1-T_1}$ for the isolated $[Cu_4Br_6]^{2-}$ anion in $[N(C_3H_7)_4]_2[Cu_4Br_6]$, one can estimate energies for self-trapping of 0.17 eV (STE1) and 0.53 eV (STE2).

Supporting Note 2.
Fluence-Dependent Transient Absorption Experiments

In Supporting Figure 2, we present the results of broadband transient absorption measurements for an $[N(C_3H_7)_4]_2[Cu_4Br_6]$ thin film in which the fluence of the 266 nm pump beam was varied systematically. The pump powers were 49, 99 and 237 µW, from which we estimate initial S_1 number densities of 6.6 x 10^{17}, 1.3 x 10^{18} and 3.2 x 10^{18} cm^{-3}, respectively. Importantly, the shape of the kinetics recorded at different wavelengths was largely independent of the pump fluence (panels d and e) and could be fitted by the same set of time constants. Also, a test for second-order behavior did not provide clean straight lines (panel f). These findings indicate that higher-order processes, such as triplet–triplet annihilation (TTA), do not play a significant role under our excitation conditions. In panel c, we observe a beginning saturation of the signal at the highest pump power. Therefore, the small spike in the blue transient at early times in panel d possibly originates from the relaxation of a two-photon excited sub-population or a change in the cooling time constant.

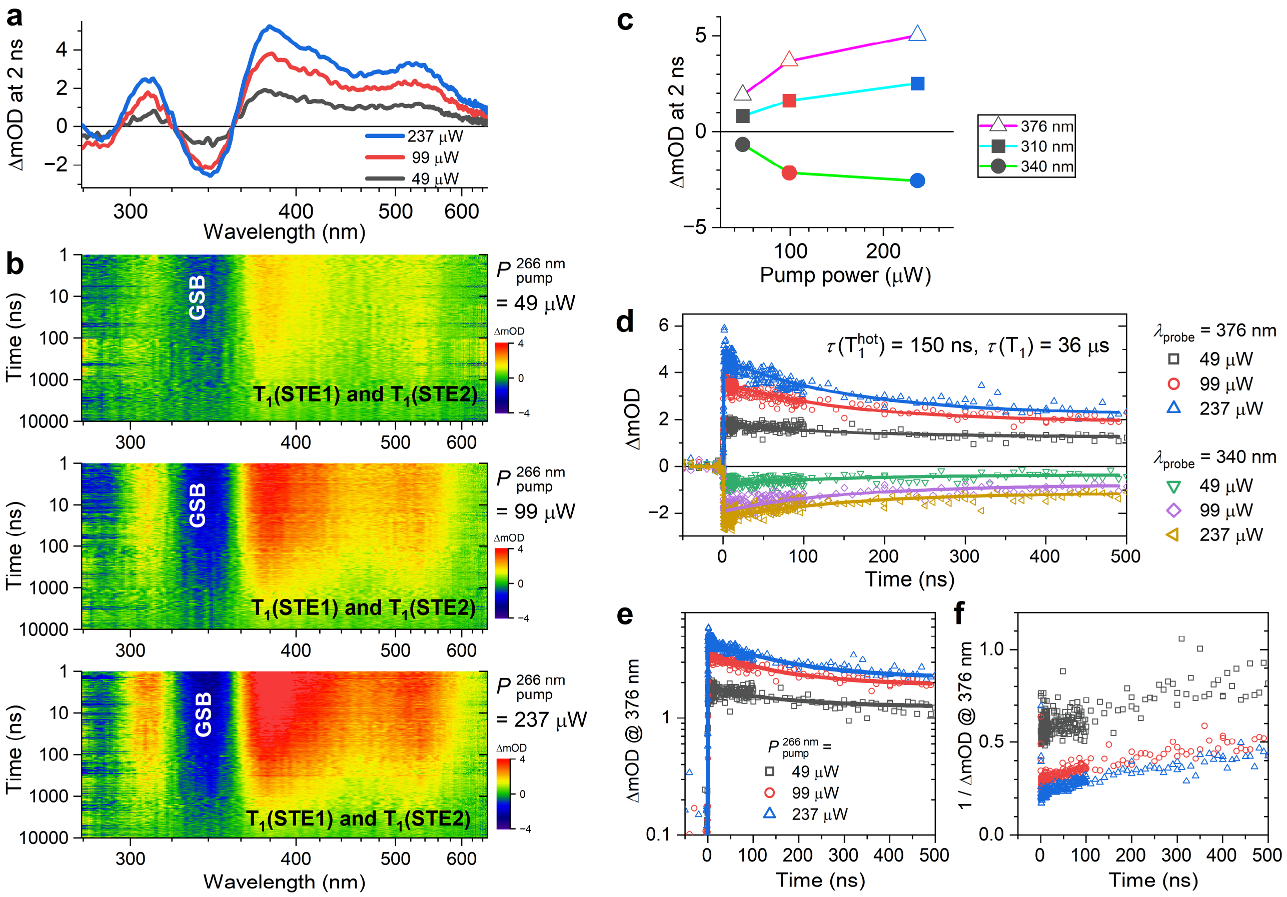

Supporting Figure 2. Results of transient absorption experiments of an $[N(C_3H_7)_4]_2[Cu_4Br_6]$ thin film at different fluences of the pump beam (λ_{pump} = 266 nm). (a) Transient spectra at 2 ps for excitation with 49, 99 and 237 µW. (b) Contour plots of the transient spectra for the three different pump powers. (c) Transient absorption at 2 ns as a function of pump power for the probe wavelengths 310, 340 and 376 nm. (d) and (e) Fluence-dependent kinetics at the probe wavelengths 340 and 376 nm including biexponential fits plotted on a linear and semilogarithmic scale, respectively. (f) Plot of the inverse absorbance as a function of time for the three experiments at different pump power.

Supporting References

[1] A. Alkauskas, M. D. McCluskey, C. G. Van de Walle, *J. Appl. Phys.* **2016**, *119*, 181101.
[2] A. Merker, M. Scholz, M. Morgenroth, T. Lenzer, K. Oum, *J. Phys. Chem. Lett.* **2021**, *12*, 2736-2741.
